# Postherpetic neuralgia in the elderly

**DOI:** 10.1111/j.1742-1241.2009.02089.x

**Published:** 2009-09

**Authors:** R W Johnson, J McElhaney

**Affiliations:** 1 University of BristolBristol, UK; 2 Division of Geriatric Medicine, University of British ColumbiaVancouver, BC, Canada

## Abstract

Postherpetic neuralgia (PHN) is the most common complication of herpes zoster (HZ) or ‘shingles’ and affects a significant proportion of HZ patients with the disease, with the elderly being most frequently and seriously affected. Characterised by various types of pain (constant, intermittent and stimulus evoked) that persist between 3 months and many years after the resolution of the HZ rash, PHN can have a severe impact on the patient’s quality of life and functional ability. PHN remains highly resistant to current treatments. In this review, we discuss the epidemiology, clinical features and management of PHN in the elderly and the potential of vaccination against varicella zoster virus as a means to prevent HZ, and thus decrease the incidence and severity of PHN.

**Review Criteria**

Information on postherpetic neuralgia was gathered via a search for relevant primary and review literature in the PubMed database using the search terms ‘postherpetic neuralgia’ or ‘PHN’, and a wide variety of key areas of interest (e.g. ‘epidemiology’, ‘management’ and ‘pain’). This was then supplemented by references found within relevant selected papers and by the clinical experience of the authors.

**Message for the Clinic**

Postherpetic neuralgia (PHN), the most common complication of herpes zoster, may have a serious impact on quality of life and functional ability, particularly in the elderly. More effective therapies are needed as PHN is often refractory to current clinical management strategies. A prophylactic vaccine against varicella zoster virus represents a promising clinical approach to limit the debilitating complications of herpes zoster, including PHN.

## Introduction

After a primary varicella infection (chickenpox), the varicella zoster virus (VZV) can remain persistent but clinically latent in the sensory nerve ganglia for many years before being reactivated and becoming manifest clinically as herpes zoster (HZ) or ‘shingles’ (1–4). HZ is characterised by a skin rash that is localised to the sensory region of the affected ganglia and is often preceded or accompanied by acute pain or itching. Pain may persist for months or even years, and this postherpetic neuralgia (PHN) is the most common and debilitating complication of HZ. There is currently no consensus definition for PHN. However, data that identify three distinct phases of pain (acute herpetic neuralgia, subacute herpetic neuralgia and chronic pain or PHN) in HZ suggest that PHN might be best defined as pain lasting at least 3 months after resolution of the rash (5–7). PHN is associated with significant loss of function and reduced quality of life, particularly in the elderly (8), and is highly resistant to treatment. Management of PHN is currently limited to antiviral therapy addressing the underlying cause (VZV infection) and analgesics for the symptomatic treatment of acute pain. This review focuses on the epidemiology, clinical features, management and prevention of PHN in the elderly.

## Epidemiology of HZ and PHN

Herpes zoster is a relatively common condition; the incidence of acute HZ in the general population in Europe ranges from about 1.2 to 5.2 per 1000 person-years (annual events per 1000 population) (9–15). There is a strong correlation between the incidence of HZ and increasing age, with a marked rise in incidence at the age of 50–60 years and older (9,10,14). The lifetime risk of HZ is estimated to be up to 25% in the general population, thus one in four people may experience HZ in their lifetime. This risk rises to 50% in those aged > 85 years (16,17).

Herpes zoster is more common in individuals with immunosuppression subsequent to HIV infection or prophylaxis to avoid rejection of organ transplants, certain malignancies or treatment thereof, or treatment for inflammatory or autoimmune disease (18).

Of patients with HZ, approximately 14% will develop complications, the most common of which is PHN. Estimates of the prevalence of PHN vary widely depending upon the definition of PHN used, the study methodology and the study population. PHN persisting at 3 months may occur in 10–20% of HZ patients aged > 50 years (15,19–21). Other less frequently occurring complications may be neurological (e.g. anaesthesia in the affected dermatome, motor paresis), ophthalmic (e.g. pan ophthalmitis, keratitis, scleritis, uveitis and loss of corneal sensation), cutaneous (e.g. scarring, bacterial superinfections of HZ lesions), visceral, or may involve systemic dissemination of the virus (e.g. cerebral vasculitis, pneumonia) (22,23).

Age is the predominant predictor of PHN (24,25). In a UK primary-care study, the prevalence of PHN (3-month definition) increased markedly with age: from 8% at age 50–54 years to 21% at age 80–84 years (15). Other risk factors for PHN include prodromal pain, severe acute pain, rash severity and, in some cases, concomitant disease (26,27). There is also evidence that PHN is more common in women than in men (15,28).

Throughout Europe, the elderly population is expanding rapidly: it is estimated that by 2040 the population aged > 80 years will have doubled to almost 10% of the total population (29). An upward trend in the incidence of HZ and particularly in PHN that has already been observed is thus expected to continue (30). As it is the elderly who sustain the greatest burden caused by PHN, in terms of pain and suffering as well as medical costs (31), this demographical shift will represent a considerable burden on the healthcare and social system.

## Clinical features of PHN

Postherpetic neuralgia can manifest as different kinds of pain. It may present as constant pain, characterised by burning, aching or throbbing; as intermittent pain, including stabbing and shooting pain; or as stimulus-evoked pain, such as allodynia (the experiencing of pain after a normally non-painful stimulus, such as a cold breeze or a light touch from clothing) (16).

While PHN will resolve in most cases, it may persist in some patients for an extended period. A long-term study demonstrated that 9% (11 out of 12 of whom were aged > 51 years) still had pain at 1 year after resolution of the rash (32). In a recent randomised, double-blind, placebo-controlled, multi-centre study in the USA, the Shingles Prevention Study, in the placebo group, in people aged ≥ 60 years, 80 out of 642 (12.4%) had PHN at 3 months and in 41% of these cases PHN persisted for at least 182 days (19). Other studies have reported that PHN can last for years (21).

The negative impact of PHN on the quality of life can be similar to that caused by life-threatening diseases or serious psychological conditions. PHN can have a significant effect on many aspects of a patient’s life, causing chronic fatigue, sleep disorders, difficulty in concentrating, depression and anxiety, anorexia, loss of bodyweight and social isolation (33,34). PHN can interfere with basic and essential activities of daily living, such as dressing, bathing, mobility, travelling, shopping, cooking and housework, thus considerably impairing an individual’s functional ability and, in some cases, causing an active member of the community to become relatively inactive and housebound (16,34). The more severe the pain experienced, the more significant the impact on quality of life; in a study of 50 patients with HZ, those with pain scores ≥ 4 out of 10 experienced the greatest interference with daily living, including general activity, work, sleep and enjoyment of life (35).

With more than 20% of the population of the European Union already aged > 60 years (36) and continuing increases in life expectancy predicted, attention is being focused on the concept of ‘healthy aging’. Prevention of HZ and the disability associated with PHN would help people to remain active in old age, compressing the period of senescence to nearer the end of life (37,38).

## Management of HZ and PHN

The delayed or atypical presentation of HZ often prevents timely treatment; studies suggest that only 25–50% of patients may receive antivirals at an early stage (32,39). Antiviral agents (acyclovir, valaciclovir, famciclovir and brivudin) can alleviate acute pain and reduce the risk of long-term pain in patients with HZ (40); however, it is unclear to what extent they reduce the incidence of prolonged PHN. Corticosteroids, given either orally (41,42) or as a single epidural injection (43), have also shown beneficial effects on acute pain, but they do not prevent PHN (44).

Optimal pain control is difficult to achieve with currently available medications, and no single treatment is completely effective for all patients; in clinical practice, the combinations of analgesic drugs used usually only achieve partial pain relief (45). Moreover, the complex and heterogeneous nature of the mechanisms that contribute to PHN suggest that adequate symptom relief by a single agent is unlikely (46). The benefits and disadvantages of the main PHN therapies are summarised in Table 1.

**Table 1:** The beneficial effects and potential limitations of common treatments for postherpetic neuralgia

Class	References	Treatment	Benefits	Limitations
**Systemic**				
Tricyclic antidepressants	45–48	In general	Effect on pain	Poor AE profile: anticholinergic AEs (drowsiness, dry mouth, constipation, increased appetite)
				Rare: blurred vision, urinary retention, glaucoma exacerbation, mood change
		Amitriptyline	Effect on pain in some patients	
		Nortriptyline	Effect on pain in some patients	
		Desipramine	Less sedating than other TCAs. Less toxic side effect profile than amitriptyline	
		Maprotiline	Effect on pain in some patients	Lower efficacy than amitriptyline, poor side effect profile
Anticonvulsants	40,45,47,48	Gabapentin	Higher benefit-to-AE ratio than TCAs. Effect on pain, sleep interference, positive effect on mood, QoL	AEs: somnolence, dizziness, ataxia, peripheral oedema, infection (all usually minor)
				Caution required in patients with myasthenia gravis or impaired renal function
		Pregabalin	Effect on pain, sleep	Mild-to-moderate dizziness, somnolence, headache, dry mouth
		Carbamazepine		Little/no pain benefit
				Confusion and sedation in elderly patients
		Lamotrigine	Some evidence of neuropathic pain benefit	Dermatological complications more severe than other drugs in this class
Opioids	40,45,47,48	In general	Effect on pain. May be preferable to TCAs	Stronger opioids are recommended to be administered in a specialist clinic only and are therefore not first-line therapy
				AEs: respiratory depression, constipation, sedation, nausea, vomiting, delirium, dependence
		Morphine	Effect on pain	
		Oxycodone	Effect on pain	
			May provide significant pain relief	
		Levorphanol	Effect on pain	
		Tramadol	May provide satisfactory pain relief	Cannot be administered with antidepressants
NMDA antagonists (antagonists of the *N*-methyl d-aspartate receptor)	45,47,48	Ketamine	May be effective in some patients (poor evidence available)	AEs: itching, painful induration at injection site, nausea, fatigue, dizziness, psychodysleptic/cognitive effects
		Dextromethorphan		Not effective in PHN
Amino-amide	45,48	i.v. Lidocaine		No significant effect on pain
				AEs: mild nausea, light-headedness
**Topical**				
Amino-amide	47,48	Lidocaine 5% patch	Effect on pain May be beneficial in allodynia Minimal systemic uptake Available in self-adhesive patch	Some local irritation possible
Capsaicinoid	45,48	Capsaicin	Effect on pain	Pungent
				Burning sensation

AE, adverse event; QoL, quality of life; TCA, tricyclic antidepressant; NMDA, *N*-methyl-D-aspartate; PHN, postherpetic neuralgia.

A recent meta-analysis has demonstrated evidence of analgesic efficacy in established PHN for several orally administered therapies: tricyclic antidepressants (TCAs), opioids (including tramadol) and anticonvulsants (gabapentin and pregabalin) (45). TCAs and gabapentin are recommended as first-line therapy for this condition, being almost equally efficacious in the treatment of PHN (40,45,47), although TCAs are associated with more minor adverse events than gabapentin and are more likely to interact with other drugs (48), a particular concern in elderly patients who are likely to be using concomitant medications for chronic diseases. Pregabalin, an analogue of gabapentin, has been shown to significantly decrease pain and improve sleep in randomised, placebo-controlled trials (49,50). Although opioids also appear to be effective in controlling neuropathic pain, the age and frailty of the patient limit the prescription of such drugs known to produce moderate-to-severe side effects such as nausea and constipation (40).

Topical application of lidocaine is a well-tolerated supplementary treatment for PHN, and patches containing lidocaine are an approved treatment in the United States and Europe (51,52). There is conflicting evidence for the use of topical lidocaine (patches or gel) or a eutectic mixture of local anaesthetics (EMLA®, AstraZeneca, Miami, FL, USA) in the treatment of PHN with allodynia; while a recent systematic review found insufficient evidence to support its use (53). Previous reviews have concluded that lidocaine should be a first-line adjunctive treatment in this context (45,52,53). The topical use of capsaicin has been shown to provide significant pain relief in two randomised trials, although patient response can be delayed and discomfort and burning sensations can limit compliance (45,53).

While not supported by evidence from randomised clinical trials, some clinicians treat PHN with peripheral, epidural, intrathecal or sympathetic nerve blockade with local anaesthetics and/or steroids. Such interventions have also been used in the treatment of HZ, but results for the prevention and treatment of PHN have been disappointing as only short-term analgesic effects have been demonstrated (43,54,55). Two studies on intrathecal or epidural administration of methylprednisolone with or without lidocaine (56,57) appear to show considerable efficacy in the intrathecal groups, although the safety of this technique remains uncertain (58). Until these studies have been replicated, such treatment cannot be recommended.

Non-pharmacological treatments for PHN include transcutaneous electrical nerve stimulation, acupuncture and other alternative therapies. While the effectiveness of such interventions has not been adequately investigated, the low risks associated with their use suggest that they may be considered in combination with conventional treatment or in older patients who fail to respond to first-line treatments (52). Behavioural therapies such as relaxation techniques have been used with positive effects (52), and the potential benefits of psychological support for depression and pain-management strategies should not be ignored (54).

Currently available treatments are thus of limited efficacy and are associated with adverse events that are poorly tolerated, especially by elderly patients. There is a need for long-term randomised-controlled clinical trials to assess the efficacy of combinations of medications and new therapies (59).

## Prevention of PHN

The increased risk and severity of HZ and PHN with advancing age are associated with an age-related decline in VZV-specific T-cells (16). VZV-specific T-cell immunity could eventually fall to below a threshold at which symptomatic VZV reactivation is likely to occur, thereby increasing the risk of HZ (60). A prophylactic vaccine that can increase VZV-specific T-cell immunity represents a promising clinical approach to limit the debilitating complications of HZ, including PHN (16,60,61). Indeed, HZ itself is associated with an increase in VZV-specific T-cells, and recurrences of HZ in immunocompetent individuals are rare (22,60). In the Shingles Prevention Study, the efficacy of a live attenuated Oka/Merck VZV vaccine in decreasing the incidence and/or severity of HZ and PHN was evaluated in a trial involving 38,546 individuals aged ≥ 60 years (19). End-points included burden of illness (BOI) due to HZ, a measure reflecting the incidence, severity and duration of HZ-associated pain and discomfort (primary end-point) (19) and the incidence of HZ and PHN.

Vaccination markedly decreased both the morbidity associated with HZ and the incidence of PHN for a mean duration of at least 3 years (19,62). The BOI of HZ was significantly reduced in vaccine-recipients (61% reduction, p < 0.001 vs. placebo), and a significant reduction in the incidence of PHN at 3 months (67% reduction; p < 0.001 vs. placebo) was observed (19). Efficacy against HZ, a secondary end-point, was 51% (p < 0.001 vs. placebo). Although HZ vaccine efficacy was greater at age 60–70 years (64%) than at age > 70 years (38%), efficacy for reducing HZ BOI and PHN was similar in both age groups (19).

The vaccine was safe and well tolerated. There was no difference in overall mortality between the vaccine and placebo groups. The most commonly observed adverse events observed in the Shingles Prevention Study occurred at the local injection site (48% in vaccine-recipients vs. 16% in placebo-recipients; p < 0.05), while systemic adverse events occurred in 6.3% of vaccine-recipients and 4.9% of placebo-recipients (p < 0.05); headache being the most frequent reported (19).

This vaccine (Zostavax; Oka/Merck, Whitehouse Station, NJ) has been approved by the US Food and Drug Administration and by the European Medicines Agency. It is indicated for the prevention of HZ in individuals aged 60 years (US) or 50 years (EU) and older and is contraindicated in immunocompromised patients, children and pregnant women. Recent studies indicate that vaccination of an aged population is likely to be cost-effective from the healthcare-payer perspective (63).

## Conclusion

HZ and associated PHN can have a considerable impact on quality of life. PHN can be debilitating for some patients and may be a significant contributing factor to the progression of disability in older people. PHN is difficult to treat and is often refractory to traditional therapeutic approaches and standard analgesic regimens. More effective strategies for the management of HZ and related pain are essential. The prevention of HZ by vaccination has the potential to reduce the incidence and severity of PHN.
